# A *Mycobacterium avium* subsp. *paratuberculosis* Predicted Serine Protease Is Associated with Acid Stress and Intraphagosomal Survival

**DOI:** 10.3389/fcimb.2016.00085

**Published:** 2016-08-22

**Authors:** Abirami Kugadas, Elise A. Lamont, John P. Bannantine, Fernanda M. Shoyama, Evan Brenner, Harish K. Janagama, Srinand Sreevatsan

**Affiliations:** ^1^Division of Infectious Diseases, Brigham and Women's Hospital, University of MinnesotaBoston, MA, USA; ^2^Department of Veterinary and Biomedical Science, University of MinnesotaSaint Paul, MN, USA; ^3^United States Department of Agriculture, National Animal Disease Center, Agricultural Research ServiceAmes, IA, USA; ^4^Department of Veterinary Population Medicine, University of MinnesotaSaint Paul, MN, USA; ^5^Institute for Environmental HealthLake Forest Park, WA, USA

**Keywords:** *Mycobacterium avium* subsp. *paratuberculosis*, phagosome, intrabacterial pH, serine protease, Johne's disease, macrophage, acid

## Abstract

The ability to maintain intra-cellular pH is crucial for bacteria and other microbes to survive in diverse environments, particularly those that undergo fluctuations in pH. Mechanisms of acid resistance remain poorly understood in mycobacteria. Although, studies investigating acid stress in *M. tuberculosis* are gaining traction, few center on *Mycobacterium avium* subsp. *paratuberculosis* (*MAP*), the etiological agent of chronic enteritis in ruminants. We identified a *MAP* acid stress response network involved in macrophage infection. The central node of this network was MAP0403, a predicted serine protease that shared an 86% amino acid identity with MarP in *M. tuberculosis*. Previous studies confirmed MarP as a serine protease integral to maintaining intra-bacterial pH and survival in acid *in vitro* and *in vivo*. We show that *MAP0403* is upregulated in infected macrophages and MAC-T cells that coincided with phagosome acidification. Treatment of mammalian cells with bafilomcyin A1, a potent inhibitor of phagosomal vATPases, diminished *MAP0403* transcription. *MAP0403* expression was also noted in acidic medium. A surrogate host, *M. smegmatis* mc^2^ 155, was designed to express *MAP0403* and when exposed to either macrophages or *in vitro* acid stress had increased bacterial cell viability, which corresponds to maintenance of intra-bacterial pH in acidic (pH = 5) conditions, compared to the parent strain. These data suggest that MAP0403 may be the equivalent of MarP in *MAP*. Future studies confirming MAP0403 as a serine protease and exploring its structure and possible substrates are warranted.

## Introduction

A universal theme among bacteria is the ability to persist, replicate, and expand their territory despite which environment they occupy. Likewise several universal stressors are found within every environment such as changes in nutrients, pressures, temperatures, pHs, etc. Intrinsic and developed acid response mechanisms have been well-studied in enteric pathogens, such as *Salmonella* spp., *H. pylori, Shigella* spp., and *E. coli* (Gorden and Small, 1993; Castanié-Cornet et al., 2010; Valenzuela et al., 2011; Ryan et al., 2016). Bacteria and other microbe resistance to and survival in acid has allowed for the development of diagnostics and drugs as well as the understanding of host responses that regulate acid stress. For example, investigations into acid stress have elucidated host components of the mycobacterial disease process, such as the acidification of *M. tuberculosis* containing phagosomes (pH = 4.5–4.8) during infection (Sprick, 1956). It is also important to note that the first-line drug in TB, pyrazinamide, becomes active due to acidification of the mycobacteria containing phagosome (Zhang et al., 2014). It is therefore all the more surprising why few studies have explored mechanisms of acid resistance in mycobacteria. Current research in acid resistance mechanisms within mycobacteria have largely been focused in *M. tuberculosis* and show the involvement of several phosphate-sensing signal transduction systems (Ramakrishnan et al., 2016), cytoplasmic redox sensors (Saini et al., 2012; Mehta et al., 2016), proteases/peptidase (Vandal et al., 2008), lipoglycans (Shui et al., 2011), and transcriptional repressors (Healy et al., 2016) unrelated to phagosome maturation arrest. It is likely that similar acid resistance mechanisms exist in other mycobacterial species.

*Mycobacterium avium* subsp. *paratuberculosis* (*MAP*) is a unique member of the mycobacteria genus due to its ability to colonize and penetrate the intestinal epithelium (Bannantine and Bermudez, 2013), causing a progressive and chronic enteritis in ruminants termed Johne's disease (JD) (Sweeney, 2011). *MAP* successfully survives several acid exposures found in extracellular (soil and water) (Elliott et al., 2015), intrahost (stomach) and intracellular environments. While acid stress in *MAP* has been studied *in vitro* (Sung and Collins, 2003; Wu et al., 2007), no study to date has investigated potential mechanisms of acid resistance employed during the initial stages of cell infection. *MAP* preferentially infects subepithelial dome (SED) and lamina propria macrophages and localizes in the phagosome or phagolysosome that ranges in pH from 4.5 to 6.2 (Bannantine and Bermudez, 2013). Successful phagosome maturation leads to the increased destruction of engulfed pathogens or particles by a series of increasingly acidified and oxidatively stressed membrane-bound vesicle fusions and fissions (Kinchen and Ravichandran, 2008). Current studies have found that mycobacteria stall or inhibit phagolysosome biogenesis through (1) dysregulation Rab GTPases trafficked to the phagosome (Kelley and Schorey, 2003; Seto et al., 2011), (2) suppression of macrophage activation cytokines, particularly gamma-interferon (IFN-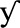) (Clemens Dl, 1995; Giacomini et al., 2001), (3) neutralization of reactive oxygen intermediates (Miller et al., 2004; Nguyen and Pieters, 2005), and (4) inhibition of vacuolar-ATPases (vATPases) crucial for establishing and maintaining acidification (Wong et al., 2011; Kissing et al., 2015). *MAP* must tightly regulate the phagosome acidification process to allow for host adaptation and recruitment of other host cells and factors necessary for pathogen survival and replication (Lamont et al., 2012), while maintaining its intrabacterial pH (pH_IB_). How *MAP* achieves this balance in the phagosome is incompletely understood.

In order to identify possible genes and mechanisms involved in acid resistance within the early stages of *MAP* infection, we conducted a microarray analysis of *MAP* in bovine MDMs treated with and without bafilomycin A1, a potent inhibitor of vATPases. We discovered an acid stress network with *MAP_RS02055* (herein referred to by its original nomenclature, *MAP0403*) serving as the central node. Computational analysis predicted MAP0403 as a transmembrane protein that shares an 86% amino acid identity to a recently characterized serine protease, Rv3671c, found in *M. tuberculosis*. A *M. tuberculosis* transposon library screen showed that a loss-of-function insertion in *Rv3671c* resulted in bacteria hypersensitivity to acid (pH = 4.5) and failure to maintain pH_IB_ in acid *in vitro* and IFN-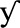 activated macrophages (Vandal et al., 2008). Furthermore, the *Rv3671c* mutant showed a growth defect using a mouse model (Vandal et al., 2008). Expression, biochemical analyses and crystallization of the Rv3671c periplasmic domain confirmed its function as a transmembrane serine protease; therefore, the protein was renamed MarP for mycobacterial acid resistance protein (Biswas et al., 2010; Small et al., 2013). Rv3671c will be referred to as MarP for the remainder of this manuscript.

This study characterizes *MAP0403* in response to intracellular and *in vitro* acid stress. We show that *MAP0403* is upregulated during initial infection in multiple cell types and exposure to extracellular acid stress. Using a *MAP* surrogate, *M. smegmatis* mc^2^ 155, expressing *MAP0403*, we demonstrate that *MAP0403* is associated with increased bacteria survival and maintenance of pH_IB_. Thus, we propose that *MAP0403* is likely a functional equivalent of *marP* in *MAP*.

## Materials and methods

### Ethics statement

All research was conducted in accordance with the University of Minnesota's Intuitional Biosafety Committee (IBC) approval protocol 0806H36901. All animal studies were conducted in compliance with the recommendations of the University of Minnesota's Institutional guidelines and approved animal care and use committee (IACUC) under approval protocol 1207A17288.

### Bacterial strains and culture

*MAP* strains K-10 (cattle isolate) and K-10 GFP (pWes4) were grown in Middlebrook medium MB7H9) supplemented with 10% glycerol, 1% oleic acid-albumin-dextrose (OADC), and mycobactin J (2 mg/L) (Allied Monitor, Fayette, MO) at 37°C with shaking at 200 rpm until the optical density at 600 nm reached 0.3. Cultures were tested for purity using *IS900* PCR (Sorge et al., 2013) and *IS1311* PCR-RFLP analyses (Amonsin et al., 2004). *M. smegmatis* mc^2^ 155 parent and pSM417-*MAP0403* and vector transformants were cultured in Luria-Bertani (LB) Lennox broth at 37°C with shaking at 200 rpm. Hygromycin (100 μg/mL) or kanamycin (50 μg/mL) was supplemented to LB and MB7H9, respectively, when appropriate.

### Mammalian cell culture

Unless otherwise noted, all mammalian cell culture and experiments were conducted at 37°C in a humidified chamber containing 5% CO_2_. MDMs from JD-free dairy cows were elutriated using a 58% percoll gradient and matured in teflon wells as described in Coussens et al. (2002) and Janagama et al. (2006). MDMs were maintained in RPMI 1640 supplemented with 20% autologous serum. Bovine mammary epithelial cells (MAC-T) were maintained in Dulbecco's modified Eagle medium (DMEM) supplemented with 10% fetal bovine serum (FBS).

### Construction of *M. smegmatis* mc^2^ 155 expressing *MAP0403*

The ORF of *MAP0403* was PCR amplified from MAP K-10 genomic DNA using a high-fidelity taq polymerase and primers 0403-HF (5′ CCC AAG CTT GTG ACG CAC TCG AAT GA 3′), 0403-SR (5′ ACA TGC ATG CTC AAC TGA CGC AGG A 3′) engineered with Hind III and Sph restriction sites at the 5′ end, respectively. The amplified fragment was cloned into a restricted pSM417 vector (herein referred to as pSM417-*MAP0403* using T4 DNA ligase and electroporated (single pulse generated at 2.5 kV, 1000Ω) into competent *M. smegmatis* mc^2^ 155 cells. Competent *M. smegmatis* mc^2^ 155 were created using the method described by Goude et al. (2015). Insert orientation and sequence fidelity were confirmed by classic Sanger sequencing at the University of Minnesota's Genomics Center. A vector control strain (pSM417 alone) in *M. smegmatis* mc^2^ 155 was also created.

### *MAP* cell invasion assay

MDMs and MAC-T cells were used to study global gene expression profiles during initial MAP infection and/or *MAP0403* expression. We used established methods described by our laboratory (Zhu et al., 2008; Lamont and Sreevatsan, 2010; Lamont et al., 2012). Briefly, 2 × 10^6^ MDMs/flask were seeded into 25 cm^2^ flasks, incubated and allowed to adhere for 2 h. Successful cell attachment was confirmed by phase-contrast microscopy. Non-adherent cells were removed by washing with PBS prior to infection. *MAP* K-10 and *M. smegmatis* mc^2^ 155 expressing pSM417 and pSM417-*MAP0403* were pelleted at 500 × *g* for 15 min, separately re-suspended in RPMI 1640 containing 2% autologous serum, and passed multiple times through a syringe-driven 18 G needle. MDMs were infected separately with the above mycobacterial strains at a MOI of 10:1 for 2 h. Upon completion of incubation, cells were washed thrice with pre-warmed PBS and treated with amikacin for 2 h to remove extracellular bacteria. Fresh medium was added to 25 cm^2^ flasks and incubated for 10, 30, and 120 min. After post-infection, MDMs were again washed in PBS and were either lysed in PBS containing 0.1% Triton X-100 or collected for RNA extraction. Triton X-100 lysates underwent differential centrifugation to separate host cellular debris from bacteria cells. Bacteria were resuspended in 1.0 mL of PBS, serially diluted on MB7H9 or LB-Lennox agar, and incubated at 37°C to determine CFUs. Hygromycin (100 μg/mL) was added to LB-Lennox agar when appropriate. MAC-T cells were seeded at a density of 2 × 10^4^ cells/well in a 24 well polysterene plate and allowed to reach 80% confluence. The same invasion method and post-infection processing as MDMs were applied to MAC-T cells with the exception that the initial infection time point was 3 h. Each time point and condition were conducted in biological triplicates. Each experiment was replicated three times.

### Phagosome acidification blocking assay

MDMs and MAC-T cells were separately seeded at 2 × 10^4^ cells/well in a 24 well plate containing No. 1.5 glass cover slips. *MAP* invasion using *MAP* K-10(pWes4)-GFP was conducted using the same conditions described above with the exception of an 1 h pre-incubation step with 50 nM of bafilomcyin A1 (A.G. Scientific Inc., San Diego, CA). Phagosome acidification was determined by a LysoTracker staining method (Lamont et al., 2012). Upon the final 30 min of each post-infection time point, 25 nM of LysoTracker Blue DND-22 (Invitrogen, Carlsbad, CA) was added to cell medium. Cells were subsequently washed thrice in Dulbecco's PBS, incubated in pre-warmed Deep Red CellMask plasma membrane stain (2.5 μg/mL) (Invitrogen, Carlsbad, CA) for 5 min, and rewashed. Cells were fixed in absolute methanol for 5 min at −20°C and washed twice in ice-cold D-PBS. Slides were stored at 4°C until visualization by confocal microscopy. An Olympus FluoView 1000 upright confocal microscope (Olympus, South-end-on-sea, Essex, United Kingdom) was used to image infected and control cell slides slides with FITC, Cy5, and DAPI lasers. Z-series for each slide was taken in 1 μM steps and stacked to render a complete image. A minimum of three fields per slide were imaged. Blocking assay was repeated in triplicate for each condition.

### RNA extraction

All work surfaces and equipment were treated with RNase Away (Molecular Bioproducts, Inc., San Diego, CA). Total RNA was extracted from MAP infected MDMs treated with/out bafilomycin A1, acid treated and control bacteria using TRIzol reagent (Invitrogen, Carlsbad, CA) per manufacturer's instructions. Bacterial lysates were homogenized using a reported bead-beating method (Janagama et al., 2010). RNA samples were treated with the TURBO DNA-free Kit (Ambion, ThermoFisher Scientific, Rockford, IL) and subjected to PCR to confirm that the samples were devoid of genomic DNA. RNA quality and concentration was determined by measuring the 260/280 ratio on a NanoDrop ND 1000 spectrophotometer (Nanodrop Products, Wilmington, DE).

### RNA processing and labeling

RNA extracted from *MAP* K-10 infected MDMs treated with/out bafilomycin A1at 30 min p.i. were processed and hybridized as described (Janagama et al., 2010). Briefly, total RNA treated with DNase was processed to remove host RNA and the 16S ribosomal RNA using MICROBEnrich and MicrobExpress kits (Ambion, Thermo Scientific, Rockford, IL) as specified by manufacturer. Microbial RNA was amplified using the MessageAmpII Bacteria kit for prokaryotic mRNA per manufacturer's instructions (Ambion, ThermoFisher Scientific, Rockford, IL). Labeled (Cy3 or Cy5) DNA was produced from microbial mRNA using the Superscript Plus Direct cDNA labeling system (Invitrogen, Carlsbad, CA) with aminoallyl-dUTP followed by a coupling of the aminoallyl groups to either Cyanine-3 or Cyanine-5 (Cy-3/Cy-5) fluorescent molecules. cDNA reactions were pooled by treatment group to obtain a sufficient concentration of labeled DNA. Effective labeling was achieved by incubating the aminoallyl-dUTP coupled cDNA with the dye for 2 h at room temperature (RT). Labeled cDNA was hybrized to 70-mer oligonucleotide microarray slides (National Animal Disease Center, Ames, IA) overnight at RT, washed in microarray buffer and scanned using the HP Scanarray 5000 (Perkin Elmer Inc., Waltham, MA). All images were stored. Microarray experiments were repeated three times. Raw microarray data files have been submitted to NCBI Gene Expression Omnibus (GEO) (Edgar et al., 2002) and are accessible through GEO series accession number GSE84708.

### Microarray data analysis

All microarray experiments were conducted using the minimal information about a microarray experiment (MIAME) guidelines. Microarray image analysis software, BlueFuse (BlueGnome Ltd., Cambridge), was used to extract numeric data from stored microarray images. Normalization by global LOWESS was performed and expression data was analyzed by GeneSpring GX 10 (Agilent Technologies, Foster City, CA). Normalized ratios were reported as fold change. Differentially expressed genes (DEGs) were cross-referenced to the *MAP K-10* genome and the remaining mycobacterial genomes listed in National Center for Biotechnology Institute (NCBI) using Basic Local Alignment Search Tool (BLAST) alogorithm. *MAP* gene networks were analyzed by the STRING database (http://www.string-db.org).

### Quantitative real time PCR

One-step qRT-PCR was performed using QuantiFast SYBR Green One-Step QRT-PCR mix (Qiagen, Valencia, CA) in a LightCycler 480II (Roche, Madison, WI) with corresponding software. The following program was used: 50°C for 10 min, 95°C for 5 min (activation), 95°C for 10 s (denature), and 95°C for 15 s, 60°C for 1 min repeated for 40 cycles (PCR amplification). Primers used for qRT-PCR were designed using Primer 3 software (http://frodo.wi.mit.edu/primer3/) and are listed in Table S1. Fold changes were calculated using the 2^−ΔΔCt^ method (Livak and Schmittgen, 2001). The value of the housekeeping gene, *secA*, was normalized to untreated bacteria cells. All samples were conducted in technical triplicates.

### Measurement of *in vitro* acid treatment

*MAP* K-10 (OD_600_ = 0.3) in MB7H9 was vortexed for 5 min and passed 10 times through a sterile 18 G syringe-driven needle to disperse bacterial clumps. The culture was allowed to stand for 5 min to facilitate the sedimentation of bacterial clumps. The upper two-thirds of the culture (containing a single cell suspension) were used in all subsequent experiments. Acidity of test cultures was adjusted to a pH of 5 by 2N HCl, while control cultures were maintained at a pH of 6.6 ± 0.2. All cultures were mixed and allowed to rotate at 37°C for 10, 30, and 120 min. Each condition was conducted in biological triplicates. All experiments were repeated three times. CFUs were determined by plating serial dilutions of the suspension in duplicate on MB7H9 agar.

### Measurement of intrabacterial pH of carboxyfluorescin labeled *M. smegmatis* mc^2^ 155-pSM417-*MAP0403*

Log-phase *M. smegmatis* mc^2^ 155 parent and pSM417-*MAP0403* transformants were labeled with 5(6)-carboxyfluorescein N-hydroxysuccinimide ester (5(6)-CFDA) (Sigma, St. Louis, MO) as described in Gaggìa et al. (2010). Briefly, 5.0 mL of bacterial cells were harvested by centrifugation at 13,000 rpm for 5 min, resuspended in 980 μl of filter (0.45 μM pore size) sterilized citric acid–phosphate-buffer (pH 7.0) supplemented with 10 μL of 1 M glucose and 10 μL of 5(6)-CFDA, and incubated for 1 h at 37°C. Upon completion of incubation, cells were sedimented at 13,000 rpm for 5 min, washed thrice in phosphate buffered saline (PBS), and resuspended in LB broth (pH = 7). A standard curve relating fluoresce with decreasing pH was established by irreversible membrane permeablization of *M. smegmatis* mc^2^ 155 parent using ethanol (63% v/v) incubation at 37°C for 30 min. Bacteria were pelleted as described above, resuspended in 5 mL of LB broth adjusted to pH range of 4–8, and incubated at 37°C for 30 min. to equilibrate pH_IB_ to medium pH. Fluorescence at each pH was measured using a SpectraMax 2000 spectrophotometer (492 nm excitation, 517 nm emission) (Molecular Probes, San Diego, CA) and the mean fluorescence units were plotted against pH. Acid treatment for parent and pSM417-*MAP0403* strains was repeated with the exception of irreversible membrane permeabilization. Fluorescence units were compared against the standard curve to determine pH_IB_. The experiment was thrice repeated.

### Statistical analysis

The results pertaining to relative fold changes and CFUs were analyzed by two-way analysis of variance (ANOVA) with Bonferroni correction in Graphpad Prism software (GraphPad Software, La Jolla, CA). *P* < 0.05 were considered to be statistically significant. Fitness of *M. smegmatis* transformants in MDM infection is shown as a box-whisker plot to demonstrate the actual distribution of observed CFUs.

## Results

### A predicted serine protease network is expressed during phagosome acidification

**Figure 1:**
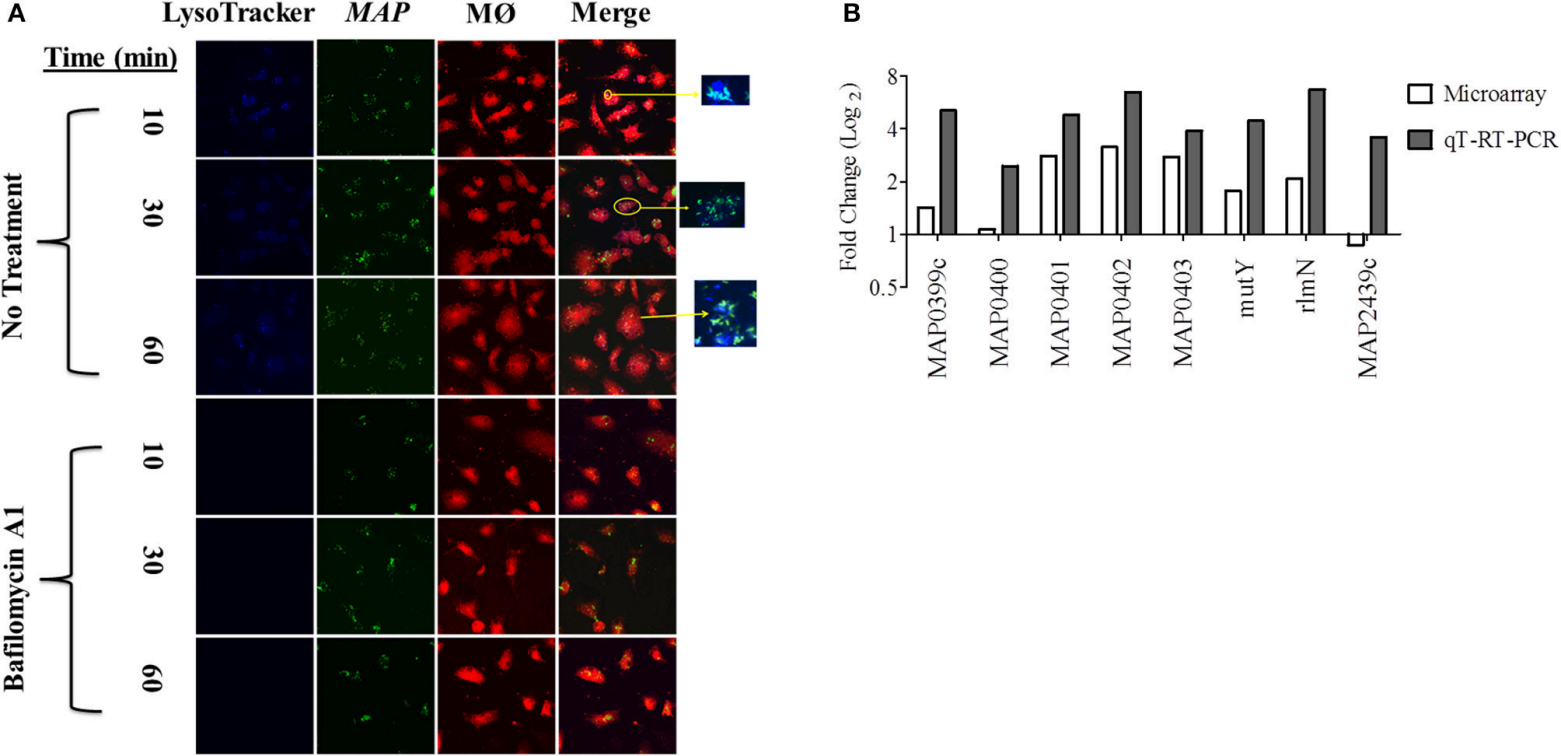
**An acid stress network corresponds with phagosome maturation in MDMs. (A)** MDMs were infected with *MAP* K-10 (pWes4) GFP and analyzed for phagosome acidification at 10, 30, and 60 min post-infection using LysoTracker Blue (above panel). Phagosome acidification was observed at 10–60 min. Acidification was validated by bafilomycin A1 inhibition of vascular ATPases (below panel). **(B)** Microarray analysis comparing *MAP* K-10 infected MDMs treated with and without bafilomycin A1 treatment at 30 min identified an acid stress related network. *MAP0403*, a predicted serine protease, was the central node of the network. All genes within the acid stress network were validated by qT-RT-PCR.

In order to identify the acid stress transcriptome during initial *MAP* infection, we first sought to characterize the phagosome maturation process in bovine MDMs. *MAP* K-10 was allowed to invade MDMS for 10–120 min post-infection (p.i.) and phagosomes were subsequently assessed for acidification using LysoTracker Blue, a fluorescent, acidotropic probe, that emits upon protonation of its basic amine. We observed phagosome acidification as early as 10 min in MDMs (Figure 1A). Phagosome acidification continued for 1 h (Figure 1A); however, the acidification process completed by 2 h p.i. (*data not shown*). Phagosome acidification was validated by pre-treatment of MDMs with bafilomcyin A1, a potent vacuolar ATPase inhibitor. Bafilomcyin A1 treatment of MDMs abrogated phagosome acidification at all p.i. time points (Figure 1A).

We selected a 30 min p.i. time point to perform a microarray analysis of genes expressed during phagosome acidification in MDMs. We compared expression profiles from MDMs infected with *MAP* K-10 with and without bafilomycin pre-treatment. Microarray analysis identified a *MAP* acid stress related network composed of genes MAP0403, *MAP*_*RS20120* (*MAP3922*), *MAP_RS12430* (*MAP2439c*), *MAP_RS02045* (*MAP0401*), *MAP_RS02035* (*MAP0399c*), *rlmN, mutY*, and *nth* (Figure 1B). All genes within the acid stress network were validated by qT-RT-PCR (Figure 1B).

*MAP0403* was the center node of the acid stress network. A Basic Local Alignment Search Tool (BLAST; https://blast.ncbi.nlm.nih.gov/Blast.cgi) protein analysis predicted that MAP0403 encodes a serine protease that is 86% identical and 92% similar to MarP found in *M. tuberculosis*. MAP0403 is composed of 397 amino acids and is projected by transmembrane helix prediction software (TMHMM) to have 4 transmembrane helices at the N-terminus, similar to MarP (Figure S1A). The C-terminal portion is predicted to contain a trypsin-like protease domain and is likely localized to the periplasm. In MarP, oxidative stress triggers autocleavage of the protease domain and stabilization of the active site through reduction of disulfide bonds and subsequent activation of the domain's protease activity (Cameron et al., 1994; Vandal et al., 2008, 2009; Biswas et al., 2010). Cysteine disulfide bond prediction of MAP0403 using DiANNA ver. 1.1 web software showed a disulfide bond located at amino acid positions 214-397 (SLAPSCQKVLE–VGTGSCVS), which is also present in MarP. Further motif analysis showed that like MarP and other serine proteases, including eukaryotic chymotrypsin proteases, MAP0403 contains the conserved catalytic triad that functions at the active site of transferase enzymes (Figure S1B). Together these data suggest that MAP0403 is likely the equivalent of MarP in *MAP*. Given the role of *MAP0403* as the central node in an acid stress network and its conservation to MarP, we focused on elucidating the association of *MAP0403* with acid stress and pH_IB_.

### *MAP0403* transcription during initial cell infection is associated with increased bacterial survival

**Figure 2:**
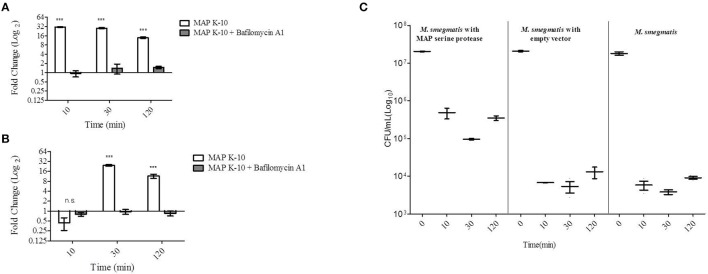
***MAP0403* is expressed in MDMs and MAC-T cells and correlates with *M. smegmatis* survival during infection**. *MAP0403* expression was analyzed by qT-RT-PCR in **(A)** MDMs and **(B)** MAC-T cells at 10, 30, and 120 min post *MAP* K-10 infection. *MAP0403* is up-regulated within 10 min post-infection but decreases by 14-fold at 120 min in MDMs. *MAP0403* expression peaks at 30 min post-infection in MAC-T cells. Similar to MDMs, *MAP0403* expression decreases at 120 min in MAC-T cells. Bafilomcyin A1 pre-treatment of MDMs and MAC-T cells decreased or abolished *MAP0403* expression. **(C)** *M. smegmatis* mc^2^ 155 parent, *MAP0403* expression strain (pSM417-*MAP0403*), and vector control [pSM417 (empty)] were allowed to invade MDMs for 10, 30, and 120 min and subsequently assessed for bacterial viability by direct plating. *M. smegmatis* mc^2^ 155 expressing *MAP0403* cells had higher rates of recovery compared to parent and vector control strains. ^***^P < 0.001, n.s. not significant.

To further understand *MAP0403* and its role in bacterial survival during early events in host infection, we analyzed *MAP0403* transcription in *MAP* K-10 exposed to MDMs treated with and without bafilomycin A1. As previously performed, *MAP* K-10 was allowed to invade MDMs for 10, 30, and 120 min p.i. and *MAP0403* transcription was analyzed by qT-RT-PCR. *MAP0403* was up-regulated by a 30-fold difference compared to control *MAP* K-10 within 10 min (Figure 2A). *MAP0403* upregulation was sustained throughout all p.i. time points; however, expression was reduced by 14-fold at 120 min (Figure 2A). Pre-treatment of MDMs with bafilomycin A1 decreased *MAP0403* expression at all p.i. times (Figure 2A). We next sought to analyze *MAP0403* transcription during infection within another cell type to determine if expression occurs only in a specific host cell. We used MAC-T cells, a mammary epithelial cell line, as a surrogate (Patel et al., 2006) for the intestinal epithelium—the first tissue encountered by *MAP* during natural infection. In a previous study, we showed that phagosome acidification of MAC-T cells infected with *MAP* K-10 occurred within 10 min and was sustained until 60 min p.i. (Lamont et al., 2012). Unlike MDMs, *MAP0403* differential expression was not detected until 30 min p.i. (Figure 2B). The 30-fold upregulation of *MAP0403* compared to the bacteria control corresponded to peak acidification in MAC-T cells (Figure 2B) (Lamont et al., 2012). *MAP0403* expression decreased by 120 min p.i. and showed a 16-fold difference compared to control (Figure 2B). As expected, pre-treatment of MAC-T cells with bafilomycin A1 abolished *MAP0403* expression at all p.i. time points (Figure 2B).

We further interrogated the role of *MAP0403* during cell infection by asking whether or not expression was critical to bacteria survival. Attempts by our laboratory to create a *MAP0403* deletion in *MAP* have been unsuccessful. Therefore to answer this question, we utilized the non-pathogenic mycobacteria, *M. smegmatis* mc^2^ 155, as a surrogate host to express *MAP0403* due to its inability to survive in macrophages past 48 h (Lagier et al., 1998; Kuehnel et al., 2001; Anes et al., 2003, 2006). It is important to note that *M. smegmatis* mc^2^ 155 contains a gene, *MSMEG_6183*, that is predicted to encode a serine protease that shares 66 and 68% amino acid identities with MarP and MAP0403, respectively. However, we found that *MSMEG_6183* is not up-regulated during MDM infection or under *in vitro* acid stress (pH = 5) compared to control bacteria at 10-120 min p.i. (*data not shown*). *M. smegmatis* mc^2^ 155 was electroporated with a plasmid expressing *MAP0403* from an Hsp60 promoter (*M. smegmatis* pSM417-*MAP0403*) or an empty vector [*M. smegmatis* pSM417(empty)]. *M. smegmatis* mc^2^ 155 parent and transformants were exposed to MDMs for 0, 10, 30, and 120 min p.i. and assessed for bacterial viability. *M. smegmatis* expressing *MAP0403* showed a greater than 1.5 log_10_ increase in CFU recovery compared to *M. smegmatis* controls within 10 min p.i. (Figure 2C). Increased recovery of *M. smegmatis* pSM417-*MAP0403* vs. controls was maintained throughout MDM infection (Figure 2C). *MAP0403* transcription was evaluated in *M. smegmatis* mc2 155-*MAP0403* in response to MDM infection (Figure S2). However, *MAP0403* expression was not differentially expressed to the control time point at 0 min (Figure S2A). This is likely due to its constitutive expression under the pSM417 Hsp60 promoter. In summary, these data provide initial support that *MAP0403* is expressed during host infection in different cell types undergoing phagosome acidification and likely aids in bacterial cell survival.

### MAP0403 transcription and association with bacterial survival and intrabacterial pH homeostasis under extracellular acid stress

**Figure 3:**
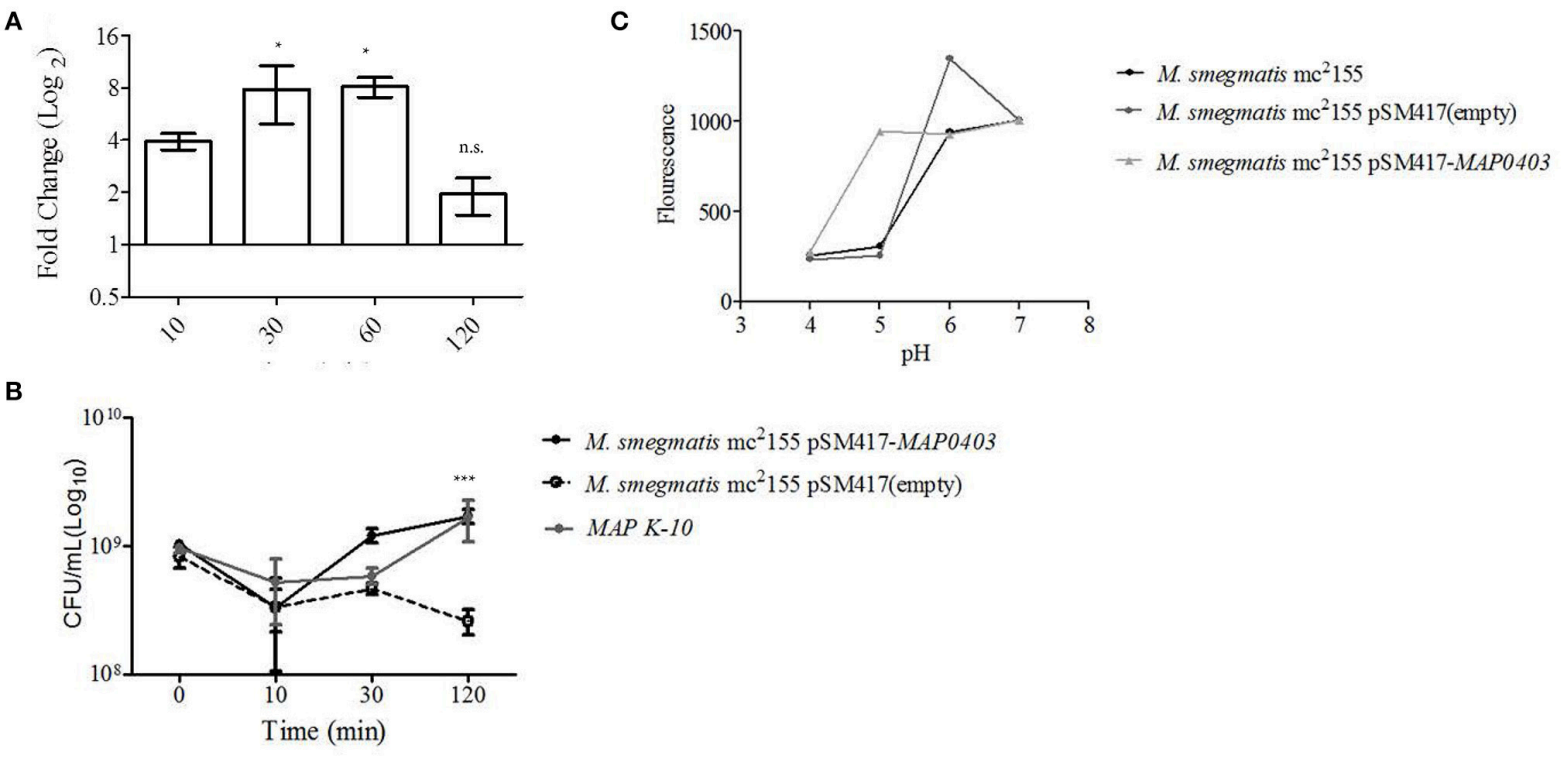
***MAP0403* aids in bacterial survivability during *in vitro* acid exposure. (A)** *MAP* K-10 was exposed to acidified (pH = 5.0) MB7H9 for 10, 30, and 120 min, recovered, and analyzed for *MAP0403* expression by qT-RT-PCR. *MAP0403* was up-regulated by 4-fold within 10 min of acid exposure and reached peak expression by 30 and 60 min. **(B)** *MAP* K-10, *M. smegmatis* mc^2^ 155 parent, *MAP0403* expression strain (pSM417-*MAP0403*), and vector control [pSM417 (empty)] were exposed to the same acid conditions as **(A)** and underwent direct plating to determine viability counts. *MAP* K-10 and *M. smegmatis* mc^2^ 155 expressing *MAP0403* cells had higher rates of recovery compared to *M. smegmatis* mc^2^ 155 parent and vector control strains. **(C)** Intra-bacterial pH measurement in *M. smegmatis* mc^2^ 155 parent and transformants. Intra-bacterial pH was measured by a fluorescent marker, 5(6)-carboxyfluroescein N-hydroxysuccinimide, in *M. smegmatis* mc^2^ 155, *MAP0403* expression strain (pSM417-*MAP0403*), and vector control [pSM417 (empty)] exposed to various pHs ranging from 4 to 7. Compared to controls, *M. smegmatis* pSM417-*MAP0403* maintained its intra-bacterial pH in acidified medium (pH = 5). ^*^P < 0.05, n.s. not significant.

Although phagosome acidification was observed in both MDMs and MAC-T cells, acid stress represents one of several host stresses against *MAP* and other factors including Reactive Oxygen Species (ROS) and Reactive Nitrogen Intermediates (RNI), etc. were likely present within the phagolysosome. Therefore, the sole contribution of acid to *MAP0403* transcription could not be determined. In order to understand the direct influence of acid stress to *MAP0403* expression and bacteria survival, we took a reductionist approach by employing acid stress *in vitro* to *MAP* K-10 and *M. smegmatis* mc^2^ 155 pSM417-*MAP0403*. *MAP* K-10 was separately incubated with acidified (pH = 5) and untreated broth (pH = 6.8) for 10–120 min and interrogated for *MAP0403* differential expression. Acid treatment resulted in a four-fold upregulation of *MAP0403* within 10 min (Figure 3A) compared to control. *MAP0403* transcription increased to ~8-fold by 30 and 60 min, but decreased by 120 min (Figure 3A).

*MAP* K-10, *M. smegmatis* mc^2^ 155 pSM417-*MAP0403*, and *M. smegmatis* mc^2^ 155 pSM417(empty) were incubated in the same conditions described above and analyzed for recovery using direct plating. Within 10 min of acid treatment, CFUs for all strains were reduced by 0.5-1 log_10_ compared to inoculum (Figure 3B). However, by 120 min of acid exposure *MAP* K-10 and *M. smegmatis* mc^2^ 155 pSM417-*MAP0403* recovered and stabilized in contrast to *M. smegmatis* mc^2^ 155 pSM417(empty), which showed a 1-fold reduction in CFUs (Figure 3B). We analyzed *MAP0403* transcription in *M. smegmatis* mc^2^155 pSM417-*MAP0403* in response to acid *in vitro* (Figure S2B). Similar to pSM417-*MAP0403* transcription during MDM infection, data showed that *MAP0403* was not differentially expressed (Figure S2B).

Lastly, we asked if *M. smegmatis* mc^2^ 155 pSM417-*MAP0403* viability during *in vitro* acid stress corresponded to maintenance of its pH_*IB*_. A 5(6)-CFDA fluorescence ratio microplate assay was used to determine pH_IB_ (Siegumfeldt et al., 1999; Gaggìa et al., 2010). A calibration curve was created to establish the link between fluorescence and pH (range 4–8; Figure S3). Increasing fluorescence was positively correlated to pH (Figure S3). *M. smegmatis* mc^2^ 155 pSM417-*MAP0403* and *M. smegmatis* mc^2^ 155 stained with 5(6)-CFDA were exposed to a pH range of 4–7 for 30 min, measured for fluorescence, and compared against the calibration curve. Both strains maintained a neutral pH_IB_ when exposed to extracellular alkaline pHs as evidenced by an increased fluorescence (Figure 3C). However, only *M. smegmatis* mc^2^ 155 pSM417-*MAP0403* was able to sustain a neutral pH_IB_ when incubated with medium acidified to pH = 5 (Figure 3C). All strains at pH = 4 failed to show a neutral pH_IB_(Figure 3C). Overall, data showed that extracellular acid exposure is one signal for *MAP0403* expression and similar to intracellular survival, *MAP0403* supports bacteria viability when met with an acid stressor.

## Discussion

Resistance and tolerance to acid due to multiple exposures to low pH have been well-studied in enteric bacteria. Unlike enteric pathogens, repeated incubations of mycobacteria with acid did not significantly increase resistance compared to controls (O'brien et al., 1996). Current studies investigating acid resistance in mycobacteria indicate that the genus has developed specific mechanisms to either persist in or neutralize acid found in multiple environments. Mycobacterial strategies for combating acid are now being elucidated. Because *MAP* and other known pathogenic mycobacteria, reside and persist in slightly acidic phagolysosomes, we sought to identify upregulated *MAP* genes involved in the early phagosome acidification process. Microarray analysis uncovered an acid stress network that contained *MAP0403* as its central node. We found that *MAP0403* is upregulated during the initial events of cell infection and *in vitro* acid stress. Furthermore, *MAP0403* is associated with increased bacterial survival and pH_IB_ homeostasis.

*MAP0403* is a predicted membrane serine protease and computational analysis of its secondary structure suggests it belongs to the chymotrypsin family. *MAP0403* contains 4 N-terminal transmembrane bound helices and a C-terminal protease domain likely located in the periplasm. In addition to *MAP0403*, the *MAP* K-10 genome contains three other hypothetical trypsin-like serine proteases: PepD (MAP_RS04650), PepA (MAP_RS18120), and HtrA (MAP_RS13035; Li et al., 2005). Information pertaining to PepD, PepA, and HtrA in MAP in the scientific literature concerns the presence of these proteins as serological markers or structure and sequence similarities with serine proteases found in enteric species (Cameron et al., 1994; Shin et al., 2010). For example, N-terminal sequencing of MAP_RS13035 determined that the gene shared 30% homology with *htrA* genes found in *E. coli, Salmonella* spp., *Brucella abortus*, and *R. henselae* (Cameron et al., 1994). PepA in *V. cholera* (Behari et al., 2001) and *E. coli* (Maurer et al., 2005) was shown to activate during low pH and may be utilized for pathogen protection against oxidative stress. White et al. demonstrated interaction of PepD with the 35-kDa cell wall antigen in *M. tuberculosis*. The authors suggest that PepD may represent a general stress response strategy for cell wall maintenance (White et al., 2011). HtrA functions as both a chaperone and protease in *E. coli* and is critical for cell survival under elevated temperatures and oxidative stress conditions (Skorko-Glonek et al., 2008). With the exception of MarP in *M. tuberculosis*, mechanisms of serine proteases in mycobacteria remain poorly understood and understudied.

MAP0403 closely resembles MarP in *M. tuberculosis* and shares an 86% amino acid conservation. The function of MarP as a serine protease integral for pH_IB_ homeostasis was recently confirmed by Sabine Ehrt and colleagues (Vandal et al., 2008). A *M. tuberculosis* transposon library screen identified hypersensitivity to acid stress at pH = 4.5 within a *marP* insertional mutant. The *marP* insertional mutant was unable to maintain its neutral pH_IB_ in IFN-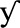 activated macrophages and after 24 h of infection pH_IB_ had lowered to 5.5. This result was also mirrored in *in vitro* acid exposure for 8 h and corresponded to 3 log_10_ reduction in viable bacteria. Complementation of the *marP* mutant restored acid resistance in *M. tuberculosis*. Attenuation of the *marP* mutant was also observed during *in vivo* infection of mice, in which 21 days p.i. resulted in a 1 log_10_ reduction compared to wild type. We also used a macrophage model to analyze *MAP0403*. Expression of *MAP0403* occurred within 10 min p.i. and corresponded with phagosome acidification (Figures 1A, 2A). Inhibition of vATPases using bafilomycin A1 diminished *MAP0403* up-regulation (Figure 2A). We further examined *MAP0403* transcription in MAC-T cells. *MAP* interacts with intestinal epithelial cells before macrophage invasion (Bannantine and Bermudez, 2013). In a previous study we showed that *MAP* induced peak phagosome acidification concomitant with calcium-dependent IL-1β secretion within 30 min p.i. to recruit macrophage recruitment to the site of infection (Lamont et al., 2012). We found that *MAP0403* expression coincided with phagosome acidification in MAC-T cells (Figure 2B). Similar to bafilomycin A1 treatment in macrophages, inhibition of vATPases abolished *MAP0403* expression. *MAP0403* upregulation was also observed in *MAP* K-10 exposed to acid *in vitro* (pH = 5) (Figure 3A).

Attempts by our group failed to grow a Δ*MAP0403* mutant. We are currently investigating other loss-of-function mutation methods such as transposonal insertion that was successfully used by Vandal et al. in *M. tuberculosis*. It is possible that *MAP0403* is an essential gene in *MAP* and that the Tetracycline-inducible system for conditional expression may be necessary (Carroll et al., 2005). Due to the lack of a Δ*MAP0403* mutant, we utilized *M. smegmatis* mc^2^ 155 expressing *MAP0403* to assess a potential role in bacteria viability. *M. smegmatis* mc^2^ 155 has a 68% amino acid identical gene, *MSMEG_6183*, to *MAP0403*. However, this gene was not upregulated in either *in vitro* acid stress (pH = 5) or MDM infection. Two scenarios exist for the absence of *MSMEG_6183* expression: (1) *MSMEG_6183* serves a different function in *M. smegmatis* or (2) conditions were not optimal or met for expression. Given that MarP and organization of the operon *MSMEG_6183* is found in is conserved within the entire Mycobacteria genus, a different function is improbable. The lack of *MSMEG*_6183 expression at our designated conditions may be a reflection of its saprophytic and fast-growing nature (*M. tuberculosis* and *MAP* are categorized as slow-growers). Further studies should simulate conditions found within soil environments. We showed that *M. smegmatis* mc^2^ 155 expressing *MAP0403* had increased CFU recovery compared to bacteria controls in both intracellular infection and *in vitro* acid stress (Figures 2C, 3B). Neutral pH_IB_ was maintained in *M. smegmatis* mc^2^ 155 expressing *MAP0403* during *in vitro* acid stress (pH = 5); however, at lower pHs the expression and control strains were unable to regulate pH_IB_ (Figure 3C). Anes et al. have noted that *M. smegmatis* undergoes three distinct phases of killing interspersed with two phases of bacterial replication during macrophage infection (Anes et al., 2006). The authors observed that the majority of phagosomes did not acidify until 8–24 h p.i. However, it was noted that bafilomcyin A1 treatment of macrophages even during early infection time points prevented killing of *M. smegmatis* suggestive that at least 20–25% of phagosomes acidify prior to 24 h p.i. The early killing of *M. smegmatis* within macrophages (phase 1) at 4 h p.i. was associated with oxidative stress. Oxidative stress is triggered by phagosome acidification (Vandal et al., 2009); therefore, the possibility remains that acidification and oxidative stress work in tandem to kill mycobacteria and both were abolished due to bafilomcyin A1 treatment. Interestingly, Biwas et al. demonstrate that *MarP* is a dual response gene and responds equally to oxidative stress (Biswas et al., 2010). Further studies should determine if (1) *MSMEG*_*6183* is upregulated in *M. smegmatis* at phase 1 killing (p.i. time point at 4 h) in macrophages, (2) whether both oxidative and acid stress are needed for *MSMEG_6183* expression, and (3) the contribution of oxidative stress to *MAP0403* expression.

While the data suggests that *MAP0403* is associated with acid stress and pH_IB_ homeostasis, the mechanism of how *MAP0403* regulates this response is unclear. MarP structural studies by Biwas et al. and Small et al. have shown that the protein (1) contains a chymotrypsin fold and disulfide bond that stabilize the protease active site for substrate binding (Biswas et al., 2010) and (2) tethering of the transmembrane helices to the periplasm is necessary for proteolysis critical for pH_IB_ during acid and oxidative stress (Small et al., 2013). The mycobacterial substrate for MarP is unknown. Biswas proposes two potential mechanisms for MarP's protection of bacterial cells in response to acid and oxidative stress (Biswas et al., 2010). The first mechanism revolves around the ability of MarP to degrade an unrelated protein, β-caesin, and suggests that MarP functions to degrade unfolded proteins found within the periplasm resultant of acid and oxidative stress. The second mechanism disassociates MarP from the stress response and states that it is essential for general cell wall maintenance and once function is lost, cell wall integrity is compromised and bacteria fail to regulate pH_IB_. Identification of the substrate bound to the active site may shed light on the correct mechanism. Comparative studies using MarP, *MAP0403*, and MarP equivalents in other mycobacteria may aid in identification or narrow potential substrates by using a “mix-and-match” method between substrates and predicted serine proteases (Madej and Kaback, 2013; Madej, 2015).

Characterization of serine proteases and other proteins necessary for acid and oxidative resistance are likely to provide a new avenue for drug and vaccine development against pathogenic mycobacteria. For example, Zhao et al. developed a whole cell screen and high throughput screen (HTS) of natural products and small, synthetic organic compounds (obtained through the National Institutes of Health Molecular Libraries Screening Center) against MarP, respectively. While the natural product screen did not yield binding to MarP (Darby et al., 2013), 9 compounds from the 324, 751 small, synthetic library reduced *M. tuberculosis* pH_IB_, inhibited MarP cleavage of β-casein, and failed to autofluoresce and interact with mammalian serine proteases (Zhao et al., 2015). Four benzoxazinones were found within the nine compounds. A specific B series benzoxazinone, BO43 (3.3 μM), decreased *M. tuberculosis'* pH_IB_ past the limit of detection by covalently acetylating the active site of MarP, which was confirmed by LC-MS/MS (Zhao et al., 2015). It is exciting to speculate that BO43 may also be active against *MAP*. Drugs targeted against serine proteases involved in pH_IB_ may be further potentiated by modulating the host response to promote acid and oxidative stress. Studies, such as the one conducted by Anes et al. focusing on how macrophages successfully kill non-pathogenic mycobacteria (Anes et al., 2006) will provide an understanding of critical responses that will inform future host related drug design. Serine proteases may ultimately provide a universal route for drug and vaccine design for pathogenic mycobacteria. Future studies confirming MAP0403 as a serine protease as well as mapping of the active site and identification of the substrate are warranted and findings will support mycobacterial treatment goals.

## Author contributions

EL, AK, FS, and EB wrote the manuscript. AK, EL, and SS designed experiments. AK and EL performed experiments. JB provided microarray slides. AK, JB, HJ, and SS analyzed microarray data.

## Funding

This study was supported by the University of Minnesota College of Veterinary Medicine Agriculture Research Station (1802-11646-AES0062027) grant awarded to SS.

### Conflict of interest statement

The authors declare that the research was conducted in the absence of any commercial or financial relationships that could be construed as a potential conflict of interest.

## Supplementary material

Figure S1 **Topology and motifs of *MAP0403*, a predicted serine protease. (A)** MAP0403, a 397 amino acid protein, is projected by the TMHMM server v. 2.0 (http://www.cbs.dtu.dk/services/TMHMM/; transmembrane helix prediction software) to have four transmembrane helices at the N-terminal. MarP (*M. tuberculosis*) and MSMEG 6183 (*M. smegmatis* mc^2^ 155), known and predicted serine proteases, respectively, are also composed of 4 N-terminal transmembrane helices. The MAP0403 serine protease domain is located at the C-terminus. **(B)** Amino acid sequence alignment of the serine protease domain belonging to MAP0403 (Q744D8), MarP (P9WHR9), MSMEG_6183 (A0R5G6) DEGP (*E. coli*), human trypsin 2, and human neutrophil esterase. Red boxes indicate the location of the conserved catalytic triad active site residues.

Figure S2 ***MAP0403* expression in *M. smegmatis* mc^2^ 155 pSM417-*MAP0403* in response to MDM infection and *in-vitro* acid stress**. *M. smegmatis* mc^2^ 155 was electroporated with pSM417 containing the ORF of *MAP0403*. *M. smegmatis* mc^2^ 155 pSM417-*MAP0403* was **(A)** infected in MDMs or **(B)** exposed to acid (pH = 5) *in vitro* for 0, 10, 30, and 120 min. *MAP0403* transcription was evaluated using qT-RT-PCR by comparing test time points (10–120 min) to control time point (0 min). *MAP0403* was not differentially expressed, which is likely due to its constitutive expression by the pSM417 Hsp60 promoter.

Figure S3 ***M. smegmatis* mc^2^ 155 intra-bacterial pH calibration curve**. Intra-bacterial pH was measured by 5(6)-carboxyfluroescein N-hydroxysuccinimide fluorescence when exposed to a pH range of 4–8.

Table S1 **Qt-RT-PCR primers used in this study**.
