# Supplementary figures and images for: A Mycobacterium avium subsp. paratuberculosis Predicted Serine Protease Is Associated with Acid Stress and Intraphagosomal Survival
Source: Front Cell Infect Microbiol. 2016 Aug 22;6:85. doi: 10.3389/fcimb.2016.00085 (PMC4992679; doi:10.3389/fcimb.2016.00085)

## Slide 1
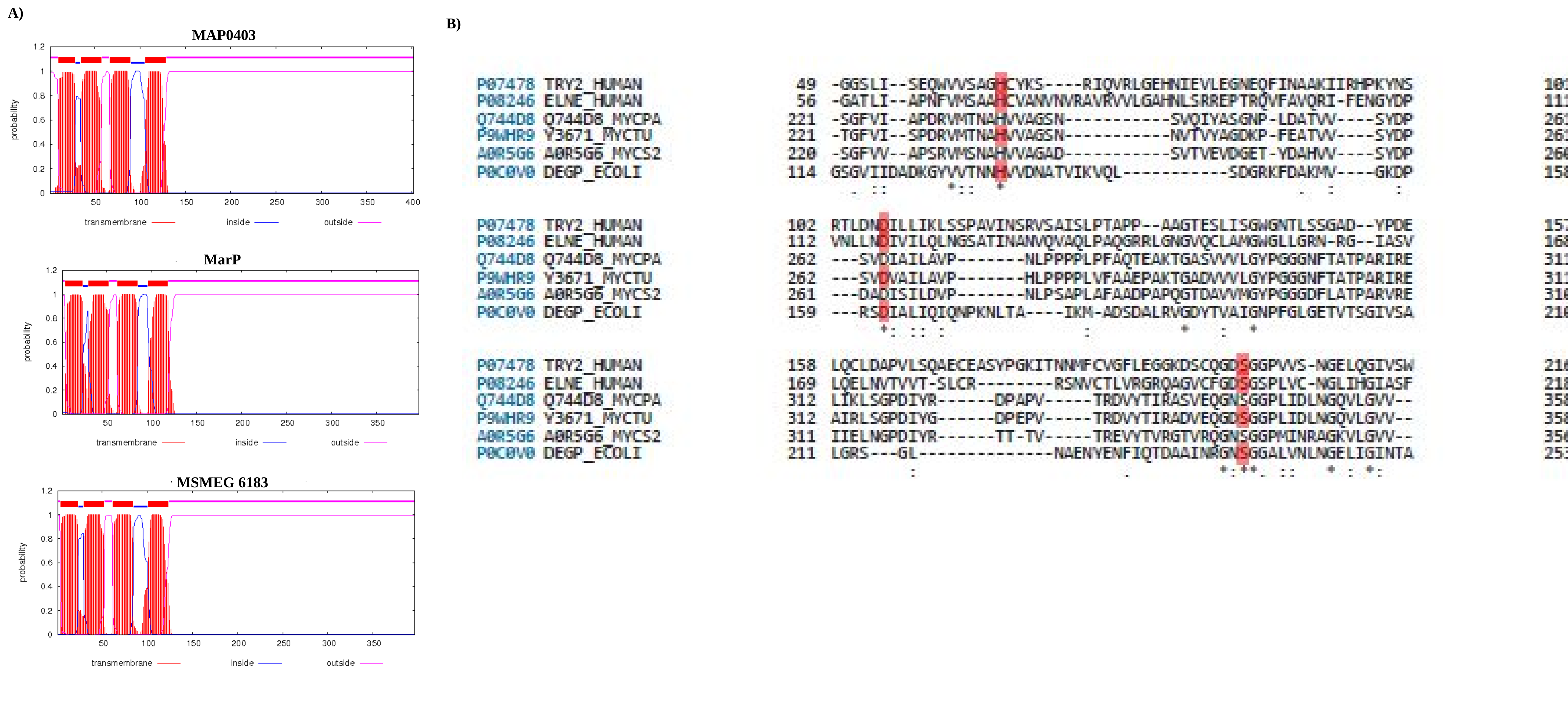

A)
B)
MAP0403
MarP
MSMEG 6183

Supplement: Figure S1 — Topology and motifs of MAP0403, a predicted serine protease. (A) MAP0403, a 397 amino acid protein, is projected by the TMHMM server v. 2.0 (http://www.cbs.dtu.dk/services/TMHMM/; transmembrane helix prediction software) to have four transmembrane helices at the N-terminal. MarP (M. tuberculosis) and MSMEG 6183 (M. smegmatis mc2 155), known and predicted serine proteases, respectively, are also composed of 4 N-terminal transmembrane helices. The MAP0403 serine protease domain is located at the C-terminus. (B) Amino acid sequence alignment of the serine protease domain belonging to MAP0403 (Q744D8), MarP (P9WHR9), MSMEG_6183 (A0R5G6) DEGP (E. coli), human trypsin 2, and human neutrophil esterase. Red boxes indicate the location of the conserved catalytic triad active site residues. [file Presentation1.PPTX]

## Slide 1
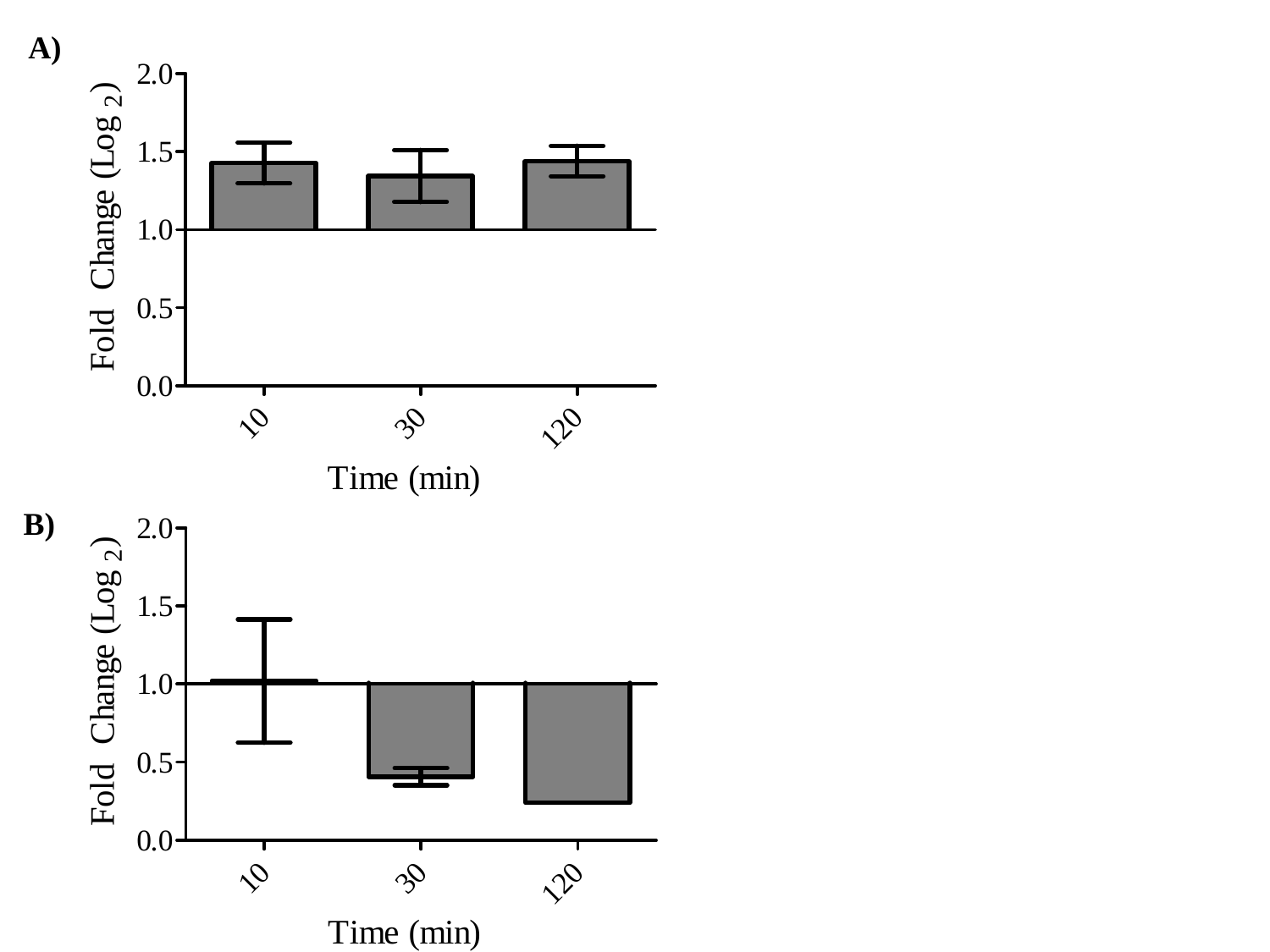

A)
B)

Supplement: Figure S2 — MAP0403 expression in M. smegmatis mc2 155 pSM417-MAP0403 in response to MDM infection and in-vitro acid stress. M. smegmatis mc2 155 was electroporated with pSM417 containing the ORF of MAP0403. M. smegmatis mc2 155 pSM417-MAP0403 was (A) infected in MDMs or (B) exposed to acid (pH = 5) in vitro for 0, 10, 30, and 120 min. MAP0403 transcription was evaluated using qT-RT-PCR by comparing test time points (10–120 min) to control time point (0 min). MAP0403 was not differentially expressed, which is likely due to its constitutive expression by the pSM417 Hsp60 promoter. [file Presentation2.PPTX]

## Slide 1
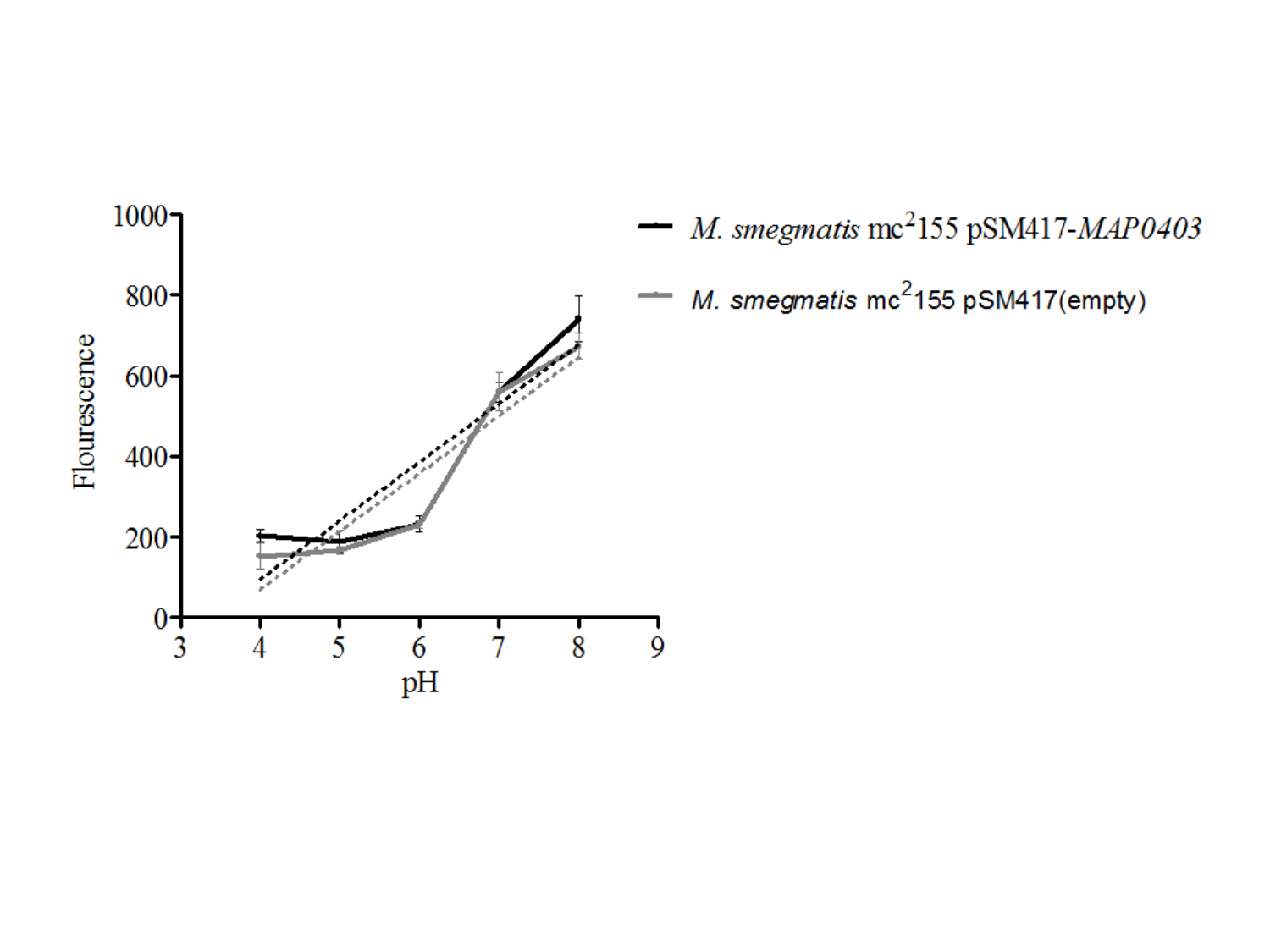

Supplement: Figure S3 — M. smegmatis mc2 155 intra-bacterial pH calibration curve. Intra-bacterial pH was measured by 5(6)-carboxyfluroescein N-hydroxysuccinimide fluorescence when exposed to a pH range of 4–8. [file Presentation3.PPTX]
