# Supplementary material for: A Mycobacterium avium subsp. paratuberculosis Predicted Serine Protease Is Associated with Acid Stress and Intraphagosomal Survival
Source: Front Cell Infect Microbiol. 2016 Aug 22;6:85. doi: 10.3389/fcimb.2016.00085 (PMC4992679; doi:10.3389/fcimb.2016.00085)
Supplement: Table S1 — Qt-RT-PCR primers used in this study. [file Table1.DOCX]

**Table S1: Qt-RT-PCR primers used in this study.**

| **Primer Name** | **Sequence and Direction** |
| --- | --- |
| MAP0469c_F | GCCCACTCATATCGGTTAC |
| MAP0469c_R | ACTCGCTGACCAGGATCT |
| MAP2439c_F | GAACAGCTCACCCGCTAC |
| MAP2439c_R | GCATCTTGTCGTAATCCAAT |
| MAP2943c_F | CAACAACCGGTGGAAGAT |
| MAP2943c_R | GTTGAGCGGTATCAGGTTC |
| MAP0399c_F | TACTCTTCGTGTGTCGTGGT |
| MAP0399c_R | CGAGGAACTCGTCGACAT |
| MAP3922_F | GTCGCTGCTGAACTACTACC |
| MAP3922_R | CCAGGTCTGAAACTGGAAC |
| MAP0404c_F | AAGACTTTTCCGAGACCATC |
| MAP0404c_R | CACGTAGGGGTCTTCCTC |
| secA_F | GGCCTGCTCCTTGAGGTT |
| secA_R | GCGCAAGGTGATCTACGC |
| MAP1575c_RT_F | CACCTGAGCTACGTCGAG |
| MAP1575c_RT_R | GATGAACGCCAGGTAGGG |
| MAP0402_F | CAACCGGCTGATGGTGTA |
| MAP0402_R | CGCTGATCGGAGGTGTC |
| MAP0401_F | GGTCGTGCTCAACTTCTGGG |
| MAP0401_R | AGGGTCGGCAGCCGAACG |
| MAP0400_F | GGATGAATCGCATTCTGG |
| MAP0400_R | AGGTGTAGCGCTTGAACA |
| SmegSigA_F | AGCGGTGAGTATCCCAGC |
| SmegSigA_R | ACGTACACCCTTTCGGTCTTA |
| MAP0403_F | TGTGGAGCAAGGCAATTC |
| MAP0403_R | CTTGGTCTCGCCGATCTT |
| MSMEG_6183_F | TGCCAGAAGGTGTTGGAG |
| MSMEG_6183_R | ACGTCGAGGATCGAGATG |
